# Sit to stand muscle power reference values and their association with adverse events in Colombian older adults

**DOI:** 10.1038/s41598-022-15757-8

**Published:** 2022-07-12

**Authors:** Robinson Ramírez-Vélez, Mikel Izquierdo, Antonio García-Hermoso, Leidy T. Ordoñez-Mora, Carlos Cano-Gutierrez, Florelba Campo-Lucumí, Miguel Ángel Pérez-Sousa

**Affiliations:** 1 Navarrabiomed, Hospital Universitario de Navarra (HUN), Navarra Institute for Health Research (IdiSNA), Universidad Pública de Navarra (UPNA), Pamplona, Spain; 2 Centro de Investigación Biomédica en Red de Fragilidad y Envejecimiento Saludable (CIBERFES), Instituto de Salud Carlos III, Madrid, Spain; 3 Laboratorio de Ciencias de la Actividad Física, el Deporte y la Salud, Facultad de Ciencias Médicas, Universidad de Santiago de Chile, USACH, Santiago, Chile; 4 Grupo de Investigación Salud y Movimiento, Programa de Fisioterapia, Facultad de Salud, Universidad Santiago de Cali, Cali, Colombia; 5 Unidad de Geriatría, Instituto de Envejecimiento, Facultad de Medicina, Hospital Universitario San Ignacio, Pontificia Universidad Javeriana, Bogotá, Colombia; 6 Grupo de Investigación en Estudios Aplicados al Deporte, Institución Universitaria Escuela Nacional del Deporte, Cali, Colombia; 7 Department of Specific Didactics, Faculty of Education, University of Córdoba, Córdoba, Spain; 8 Epidemiology of Physical Activity and Fitness Across Lifespan Research Group, University of Seville, Seville, Spain; 9 Facultad de Ciencias de la Educación, Unidad Central del Valle del Cauca (UCEVA), Túlua, Colombia

**Keywords:** Diseases, Risk factors

## Abstract

Recently, a valid method to assess lower-body muscle power based on a sit-to-stand field test (STS) has been published. Our study aimed to describe lower-body muscle power in older individuals aged ≥ 60 years and examine the relationship of muscle weakness with adverse events according to gender- and age-specific muscle weakness cut-off points. A total of 3689 Colombian older adults (57.6% women, age 69.1 ± 6.9 years) from the 2015 Survey on Health, Well-Being, and Aging in Latin America and the Caribbean (SABE) participated in this study. Lower-body muscle power normalized to body mass was estimated by the five-repetitions STS test. Anthropometric, physical performance and clinical characteristics were collected. Age-specific percentiles using the LMS method, cut-off points and association with adverse events were calculated. Lower-body muscle power was greater in men than among women (2.2 ± 0.7 vs. 1.6 ± 0.5 W·kg^−1^, respectively; *p* < 0.001) at all ages. Muscle power ranked in the 50th percentile between 2.38 and 1.30 W·kg^−1^ in men, whereas women ranked between 1.79 and 1.21 W·kg^−1^. According to the cut-off points, lower-limb muscle power < 1 standard deviation  in men was associated with having dynapenia, poor gait speed, cognitive impairment and mental, visual, hearing and memory problems. While, women were associated with having sarcopenia, dynapenia, poor gait speed, cognitive impairment, mental, hearing and memory problems, dementia and hospitalizations of > 24 h in the last year. Overall, participants with poor lower-limb muscle power had a significantly higher risk of adverse events [in men: odds ratio (OR) = 1.51, 95% confidence interval (CI) = 1.19–1.91, *p* < 0.001; in women: OR = 1.52, 95% CI = 1.27–1.87, *p* = 0.001] than their stronger counterparts. This study is the first to describe lower-limb muscle power values and cut-off points among a nationally representative sample of Colombian older adults. In men, 7 of the 14 adverse events studied were associated with lower muscle strength, whereas in women, it was 9 of the 14 adverse events.

## Introduction

In recent decades, the life expectancy in Colombia has increased for people aged 60 years and older^1^. By 2050, it is expected that the percentage of people aged 60 years will reach 30%^1^. Therefore, these predictions will represent an increase in the resources allocated to the welfare state to maintain health. It is known that aging involves senescence of the human body, thus increasing the loss of neuromuscular function^2^. Muscle weakness, poor balance and reduced walking performance are the more remarkable effects of this loss of function^2^. Such a decline is closely related to increased adverse events such as diabetes, risk of falls and hospitalization, disability, mental problems and mortality^3^.

Given the increase in the older adult population and the associated increase in neuromuscular deterioration, it would be advisable to track muscular strength. Muscular strength can be measured objectively and accurately with laboratory methods using specialized equipment such as force platforms and isokinetic equipment. However, because of the high cost of qualified professional staff and the equipment and space constraints, laboratory tests are seldom used in population-based studies^4^. In clinical practice, field tests are often the only feasible alternative because they are easy to administer, involve minimal equipment, are low cost and relatively safe, and allow many individuals to be evaluated in a relatively short time^4^.

Typically, the benchmark field test to assess muscle strength is the handgrip test, as it is a valid and straightforward assessment technique for measuring muscle strength in clinical practice^5^. Handgrip strength (HGS) reflects overall body muscle strength and all-around physical performance in older adults^5^. However, HGS is restricted to the upper segment muscles, which may not directly represent the muscle strength status of the lower limbs^6^. Therefore, it would be suitable to determine the strength of older individuals separately, especially when there is a dependence on canes or crutches. In addition, with the HGS test, we cannot assess the movement velocity. This is an essential component of strength because it refers to execution velocity^7^ and therefore to the neuromuscular demands of the movement. Consequently, HGS may not accurately represent overall strength. Fortunately, studies have been published in recent years where lower body strength is expressed in muscle power based on the sit-to-stand (STS) field test, showing it to be a valid and reliable method^8^.

The results of the Survey on Health, Well-Being and Aging in Latin America and the Caribbean (SABE, from the initials in Spanish: *SA*lud, *B*ienestar & *E*nvejecimiento) have increased our understanding of the aging process and have helped to create public policies aimed at improving the well-being of the Latin American and Caribbean populations. Recently, the normative data of HGS and the SPPB (Short Physical Performance Battery) results were published from the SABE study^9,10^, and it is mandatory to know the reference values of lower body strength in terms of power based on recent findings^8^.

Lower-body muscle power reference values in older Latin American adults and the relationship with adverse events are lacking. These reference values may help to identify target populations for primary prevention and guide population health programs, policies and priorities. For these reasons, the purpose of the present study was threefold: to describe lower-body muscle power in older individuals aged ≥ 60 years in Colombia; to identify gender- and age-specific muscle weakness cut-off points in older adults; and to investigate the relationship between disorders and adverse events and reduced lower-body muscle power by considering the muscle weakness cut-off points from the results of this study.

## Material and methods

### Study design and participants

We analyzed data from the SABE study conducted in 2015 by the Epidemiological Office of the Ministry of Health and Social Protection of Colombia (https://www.minsalud.gov.co/). The Human Subjects Committee approved the study protocol for the secondary analysis at Pontificia Universidad Javeriana (ID protocol 20/2017-2017/180, FM-CIE-0459-17) following the World Medical Association Declaration of Helsinki and Resolution 8430 for the technical, scientific and administrative standards for conducting research with humans, published in 1993 by the former Colombian Ministry of Health. Informed consent was obtained from all subjects and/or their legal guardian(s). The data used in this study were anonymized before use.

The sample included in the survey was 24,553 adults aged 60 years and above from 32 departments in Colombia. Assuming a response rate of 80%, the target sample was 30,961 individuals, calculated at the beginning. Finally, the rate was reduced to 70%, so the final sample size included was 23,694 from 244 municipalities across all departments. Details about the study design and protocol can be checked in the study published by Gomez et al.^11^.

The participants for this study were selected according to the sampling fraction concerning the general SABE sample. The following participants were excluded: individuals without any STS test, those with extreme values and participants with missing anthropometric data. A total of 3689 patients were finally included in our analysis, described in the flow chart of Supplementary Fig. S1. Having any comorbidity was not a reason for exclusion; however, older people with joint injuries in the lower limbs, unable to walk independently or diagnosed with acute neuromuscular disorders were excluded due to the characteristics of the tests.

Nurses and previously trained clinicians conducted the data collection through face-to-face interviews, and the data were computerized and used for questionnaires.

### Sociodemographic and lifestyle information

The socio-economic status was classified into six levels and categorized as low (levels I and II), medium (III and IV) and high (V and VI). Race/ethnicity was assessed by self-reporting and categorized into four categories as follows: indigenous, black “mulato” or Afro-Colombian, white and others (mestizo, gypsy, etc.). In addition, habits such as smoking cigarettes (not smoking and those who have never smoked, those who currently smoke or those who previously smoked) and alcohol consumption (drink less than 1 day per week, 2–6 days a week or every day) were evaluated. Low physical activity was measured using a form adapted to Reuben’s scale of advanced activities of daily living^12^. Thus, it was defined as a negative response to: “Can you walk, at least three times a week, between 9 and 20 blocks (1.6 km) without resting?”.

### Anthropometrics and muscle strength measurements

Before the anthropometric measurements, participants removed shoes, socks and heavy clothes. The weight scale measured body mass to the nearest 0.1 kg; height was measured using a stadiometer to the nearest 0.1 cm; and body mass index (BMI) was calculated as the ratio between body mass and height squared (kg·m^−2^). Calf circumference was measured to the nearest 0.1 cm in the standing position using a non-elastic tape measure.

Lower-limb muscle power was evaluated according to the five-repetition STS test ^13^. This test measures the time to complete five repetitions of the “sit to stand” manoeuvre on a standardized armless chair (0.45 m). The participants had to be seated on the chair without armrests, keeping the feet parallel (the legs not touching the chair), hip width apart, and the arms were hanging down loosely or on the hips. To familiarize participants with the test, two trials were performed. Then, a trained technician asked the participants to complete “as rapidly as possible” five repetitions: to stand up fully, the legs had to be straightened entirely, that is, a complete knee extension; and to sit down, the chair had to be touched by the buttocks. Time was recorded with a stopwatch to the nearest 0.01 s from the initial sitting position to the final standing position at the fifth stand. Not completed or incorrectly performed STS cycles were not counted. Relative STS mean power was calculated using the equation that was validated previously^8^, as follows: Relative STS Mean Power = 0.9 × *g* × (Height × 0.5 – Chair Height)/(Five STS Repetitions × 1/9). HGS was measured using the Takei dynamometer (Takei Scientific Instruments Co., Tokyo, Japan). Prior to the assessment, the dynamometer was calibrated to ensure proper usage and accuracy. Subjects had three attempts on each hand (alternating hands between each trial), performed with the elbow joint in full extension. The mean value was recorded as the final score of the test. Usual gait speed (m/s) was measured by walking 3 m. Participants had two attempts to walk at the usual pace starting from a standing position, with the best trial chosen.

### Metabolic disorders

Comorbidities or medical conditions were assessed by asking participants if a physician had diagnosed them with hypertension, diabetes or cardiovascular disease.

### Neuromuscular disorders

We defined sarcopenia according to calf circumference: as proposed by Rolland et al.^14^, a calf circumference of < 31 cm was considered to be sarcopenia. Dynapenia was defined according to cut-off values published by Alley et al.^15^, classifying people as weak: HGS < 26 kg for men and < 16 kg for women. Poor gait speed was defined according to the literature, with a gait speed of ≤ 0.8 m/s predicting disability and reduced overall survival^16^.

### Neurocognitive disorders

Cognition was assessed by the modified version of the Mini-Mental State Examination (MMSE), which was validated in the initial SABE studies^17^. The prevalence of mental problems was assessed with the following question: “Did a physician or nurse ever tell you that you had a neurological, mental or psychiatric problem?”. Hearing and visual problems were determined by the following questions: “Do you have difficulty hearing?” and “How would you say your far and near vision are, regardless of what glasses you wear?”^18^. Memory was examined using standardized questions^11^ developed to assess various aspects of subjective memory performance: “How would you say your memory is currently?”; “In the last two and a half years have you had problems with your memory?”; and “Compared to a year ago, how would you say your memory is?”. The answers were categorized as: “with subjective memory problems” and “without subjective memory problems”. For dementia, functional impairment was evaluated using four items of the Lawton and Brody functional scale^19^: phone use, transport use, handling medicines and management of money. The lack of functionality for doing at least two of the four activities was defined as “dependence” and an indication of dementia.

### Other adverse events

The prevalence of falls was assessed with the following question: “Have you fallen in the past year?”. Previous epidemiological studies have used this question to evaluate fear of falling^20^ and the results have demonstrated good test–retest reliability for the prevalence of falls. In addition, hospitalizations of > 24 h in the last year were recorded.

### Statistical analysis

Data are presented as mean ± standard deviation (SD) for continuous variables and as frequencies and percentages for categorical variables. Normality was assessed with the Kolmogorov–Smirnov test and probability plots. The Student’s *t*-test was applied to identify significant differences in continuous variables, and the chi-squared test was used for categorical variables. To generate gender- and age-specific normative percentiles (3rd, 10th, 25th, 50th, 75th, 90th and 97th), we applied the LMS method^21^ using LMS Chartmaker Pro (version 2.43; Institute of Child Health, London, UK). The LMS method fits smooth percentile curves to reference data by summarizing the changing distribution of gender-specific and age-group data representing skewness (L; expressed as a Box-Cox power), the median (M) and the coefficient of variation (S). The LMS method was run separately for men and women.

Finally, logistic regression models were used to compare the prevalence of disorders and adverse events according to the cut-off for lower-body muscle power originating from < 1 SD by gender and age group. Models were stratified by gender (unadjusted; see Supplementary Table S1) and fully adjusted for age, ethnicity, socio-economic status, urbanicity, BMI, smoking status, alcohol intake and physical activity “proxy”. JASP statistical package software (version 0.14.1) was used to perform statistical analysis, except for the LMS curves.

## Results

Characteristics of the study participants (anthropometric, muscle strength, sociodemographic and clinical information) are shown separately for men and women in Table 1. The mean age of the total sample (3689 participants) was 69.4 ± 7.1 years, where 2125 were women (57.6%), and 1564 were men (42.4%), with mean ages of 69.1 ± 6.9 and 69.8 ± 7.1 years, respectively. We found statistically significant gender differences (*p* < 0.05) for all anthropometric and muscle strength characteristics. For example, the mean STS power for men and women was 2.2 (0.7) W·kg^−1^ and 1.6 (0.5) W·kg^−1^, respectively (*p* < 0.001). Also, there were statistically significant gender differences for socio-economic status, urbanicity (except for rural) and ethnicity (except for black “mulato” or Afro-Colombian ethnicities). Additionally, the scarce proportions of men and women meeting the minimum required physical activity recommendations (24.8% of men and 15.2% of women) are highlighted. Regarding the prevalence of disorders, women presented more problems than men in all variables (*p* < 0.05), except in terms of memory and hearing. Regarding the incidence of other disorders, the overall sample presented the following proportions of cases: hypertension (53.3%), diabetes (16.0%) and cardiovascular disease (13.0%).

**Table 1:** Characteristics of the study participants.

Anthropometric and muscle strength	Men (n = 1564)	Women (n = 2125)	Overall (n = 3689)	P for sex
	Mean ± SD	Mean ± SD	Mean ± SD	
Age (years)	69.8 ± 7.1	69.1 ± 6.9	69.4 ± 7.1	0.003
Height (cm)	1.63 ± 0.1	1.50 ± 0.1	1.56 ± 0.1	< 0.001
Body mass (kg)	68.1 ± 12.2	62.9 ± 12.6	65.1 ± 12.7	< 0.001
Calf circumference (cm)	34.7 ± 3.4	34.6 ± 3.9	34.2 ± 3.7	< 0.001
BMI (kg/m^2^)	26.1 ± 4.1	28.2 ± 5.3	27.3 ± 4.9	< 0.001
STS time (s)	14.3 ± 4.1	15.6 ± 4.3	15.1 ± 4.2	< 0.001
STS power (W·kg^−1^)	2.2 ± 0.7	1.6 ± 0.5	1.9 ± 0.6	< 0.001
Handgrip strength (kg/body mass)	0.44 ± 0.1	0.28 ± 0.1	0.33 ± 0.1	< 0.001
Gait speed (m/s)	0.83 ± 0.27	0.73 ± 0.23	0.77 ± 0.25	< 0.001

Sociodemographic and lifestyle outcomes	n (%)	n (%)	n (%)	
**Socio-economic status**				
Level I–II	1252 (80.1)	1614 (76)	2866 (77.7)	< 0.001
Level III–IV	307 (19.6)	488 (23)	795 (21.6)	< 0.001
Level V–VI	5 (0.3)	23 (1.1)	28 (0.8)	0.001
**Urbanicity**				
Urban	1129 (72.2)	1686 (79.3)	2815 (76.3)	< 0.001
Rural	435 (27.8)	439 (20.7)	874 (23.7)	0.892
**Ethnic group**				
Indigenous	130 (9.3)	94 (5.1)	224 (6.1)	0.016
Black 'mulato' or Afro-Colombian	158 (11.2)	166 (9.0)	324 (8.8)	0.657
White	408 (29)	635 (34.4)	1043 (28.3)	< 0.001
Others^a^	709 (50.5)	953 (51.6)	1662 (45.1)	< 0.001
**Lifestyle**				
Alcohol	382 (24.4)	116 (5.5)	498 (13.5)	< 0.001
Smoking	234 (15.0)	156 (7.3)	390 (10.6)	< 0.001
Meeting PA recommendations	388 (24.8)	323 (15.2)	711 (19.3)	0.015
**Neuromuscular disorders**				
Sarcopenia	170 (11.0)	331 (15.7)	501 (13.6)	< 0.001
Dynapenia	642 (41.8)	997 (46.9)	1619 (43.9)	< 0.001
Poor gait speed	894 (58.9)	1488 (74.1)	2382 (64.6)	< 0.001
**Neurocognitive disorders**				
Cognitive impairment	159 (10.2)	277 (13)	436 (11.8)	< 0.001
Mental problems	91 (5.8)	215 (10.1)	306 (8.3)	< 0.001
Visual problems	892 (63.5)	1222 (66.3)	2114 (57.3)	< 0.001
Hearing problems	428 (27.4)	437 (20.6)	865 (23.4)	0.760
Memory	216 (13.8)	256 (12.0)	472 (12.8)	< 0.001
Dementia	77 (4.9)	168 (7.9)	245 (6.6)	< 0.001
**Metabolic disorders**				
Hypertension	714 (45.8)	1254 (59.1)	1968 (53.3)	< 0.001
Diabetes	219 (14.0)	371 (17.5)	590 (16.0)	< 0.001
Cardiovascular diseases	189 (12.1)	289 (13.6)	478 (13.0)	< 0.001
**Other adverse events**				
Falls in the last year	363 (23.2)	728 (34.3)	1091 (29.6)	< 0.001
Hospitalized > 24 h last year	154 (9.8)	243 (11.4)	397 (10.8)	< 0.001

Data are presented as mean ± SD or frequency (percentage) of participants. Student’s t-test or χ^2^ test analyzed significant differences between men and women groups.

*BMI* body mass index, *PA* physical activity, *SD* standard deviation.

^a^Others (mestizo, gitano and gipsy, etc.).

**Figure 1:**
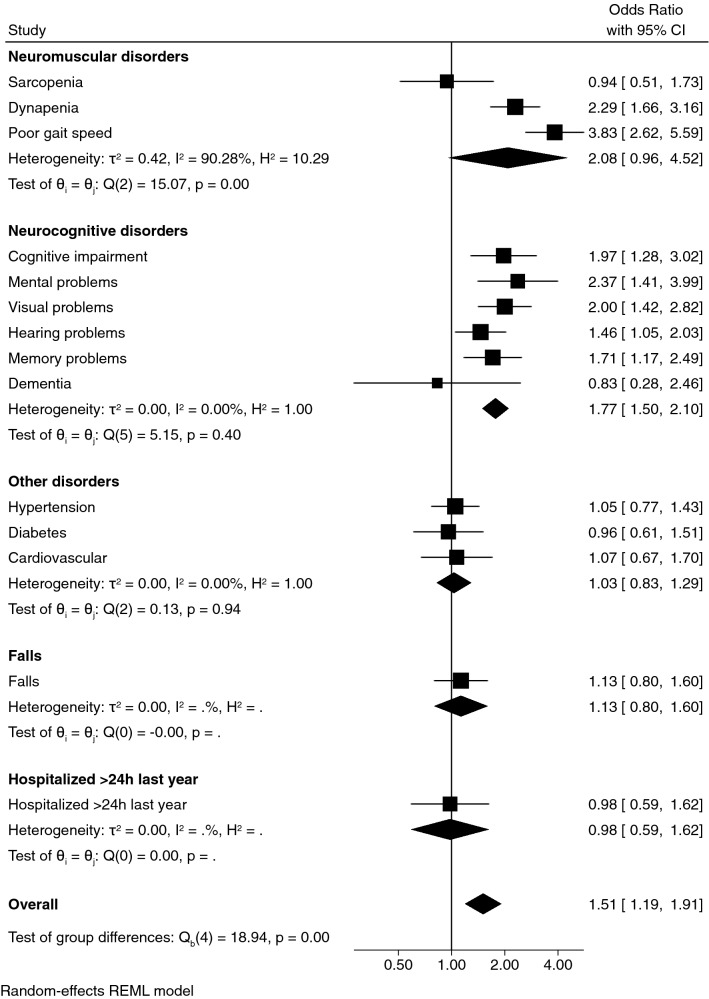
Association between poor lower-limb muscle power (W·kg^−1^) with disorders and adverse events in men. The analysis was adjusted for age, body mass index, ethnicity, socio-economic status, urbanicity, smoking status, alcohol intake and physical activity.

Table 2 and Fig. 1 show the smoothed age- and gender-specific percentiles of lower-body muscle power in men (Panel A) and women (Panel B). The percentiles show that men had better performance on the STS test than women. Men ranked in the 50th percentile between 2.38 and 1.30 W·kg^−1^, whereas women ranked between 1.79 and 1.21 W·kg^−1^. In both genders, the decrease in lower-body muscle power performance was appreciable across the age range.

**Table 2:** Smoothed age-specific and sex-specific percentile of STS relative power (W·kg^−1^) in men and women.

Sex/age group	N	L	S	Extremely low (P3)	Very low (P10)	Low (P25)	Normal (P50) M	High (P75)	Very high (P90)	Extremely high (P97)
**Men (n = 1564)**										
60–64	440	0.57	0.27	1.24	1.58	1.96	2.38	2.82	3.31	3.82
65–69	397	0.63	0.30	1.04	1.39	1.78	2.20	2.66	3.15	3.66
70–74	327	0.60	0.33	0.84	1.16	1.52	1.91	2.35	2.82	3.32
75–79	213	0.45	0.35	0.72	0.99	1.30	1.67	2.08	2.55	3.07
80–84	135	0.27	0.37	0.65	0.88	1.17	1.51	1.92	2.41	2.98
85+	52	0.23	0.42	0.57	0.75	0.99	1.30	1.72	2.29	3.06
**Women (n = 2125)**										
60–64	664	0.53	0.28	0.93	1.19	1.47	1.79	2.13	2.50	2.90
65–69	571	0.37	0.29	0.87	1.10	1.37	1.67	2.02	2.41	2.84
70–74	410	0.30	0.31	0.79	1.01	1.26	1.56	1.90	2.29	2.73
75–79	286	0.27	0.32	0.71	0.92	1.16	1.45	1.78	2.17	2.61
80–84	138	0.27	0.33	0.64	0.83	1.06	1.33	1.65	2.03	2.46
85+	56	0.26	0.36	0.55	0.73	0.95	1.21	1.53	1.91	2.35

*L* power in the Box–Cox transformation for ‘correcting’ the skewness, *M* median, *P* percentile, *S* coefficient of variation.

Lower muscle power cut-off values using < 1 SD by gender and age group are shown in Supplementary Table S2. These cut-off points ranged from 1.78 to 1.01 and 1.32 to 0.79 in men and women, respectively.

**Figure 2:**
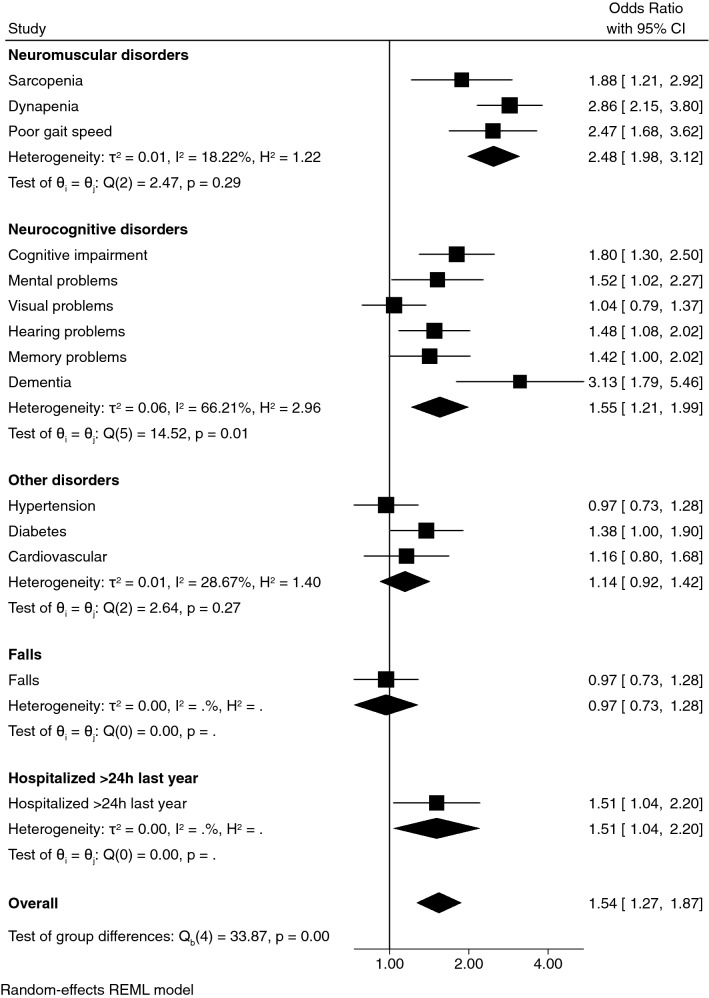
Association between poor lower-limb muscle power (W·kg^−1^) with disorders and adverse events in women. The analysis was adjusted for age, body mass index, ethnicity, socio-economic status, urbanicity, smoking status, alcohol intake and physical activity.

**Figure 3:**
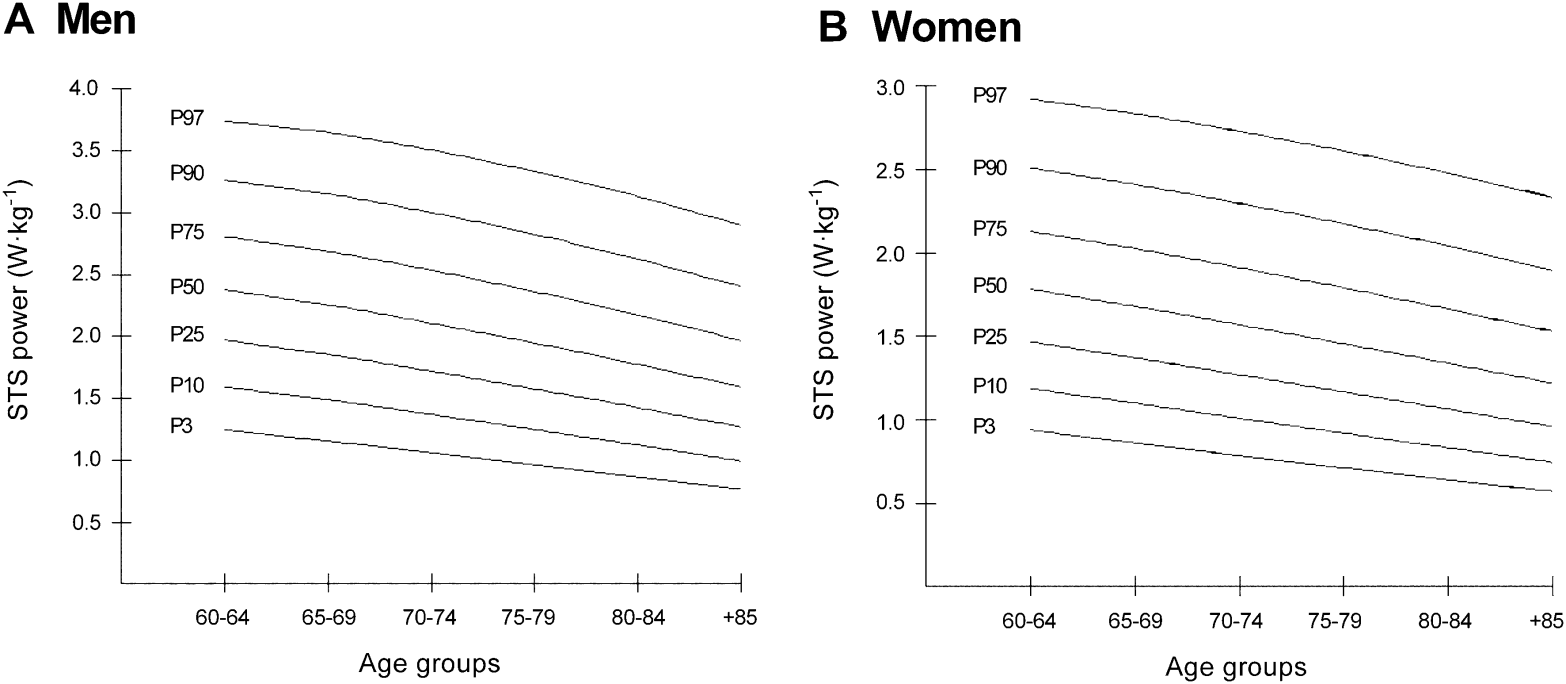
Percentile curves for lower-limb muscle power (W·kg^−1^), stratified by gender. (**A**) Lower-limb muscle power (W·kg^−1^) for Colombian men aged 60+ years, (**B**) lower-limb muscle power (W·kg^−1^) for Colombian women aged 60+ years. Percentiles showed 3th, 10th, 25th, 50th, 75th, 90th and 97th.

Figures 2 and 3 show the association between lower-body muscle power (W·kg^−1^) and disorders. Men with poor lower-body muscle power had a greater association with dynapenia (odds ratio [OR] = 2.29, 95% confidence interval [CI] = 1.66–3.16; *p* < 0.001), poor gait speed (OR = 3.83, 95% CI = 2.62–5.59; *p* < 0.001), cognitive impairment (OR = 1.97, 95% CI = 1.28–3.02; *p* = 0.002) and mental (OR = 2.37, 95% CI = 1.41–3.99; *p* < 0.001), visual (OR = 2.00, 95% CI = 1.42–2.82; *p* < 0.001), hearing (OR = 1.46, 95% CI = 1.05–2.03; *p* = 0.024) and memory (OR = 1.71, 95% CI = 1.17–2.46; *p* = 0.005) problems than healthy counterparts with strong muscle power.

Older women with poor lower-body muscle power had a greater association with sarcopenia (OR = 1.88, 95% CI = 1.21–2.92; *p* < 0.005), dynapenia (OR = 2.86, 95% CI = 2.15–3.80; *p* < 0.001), poor gait speed (OR = 2.47, 95% CI = 1.68–3.62; *p* < 0.001), cognitive impairment (OR = 1.80, 95% CI = 1.30–2.50; *p* < 0.001), mental (OR = 1.52, 95% CI = 1.02–2.27; *p* = 0.039), hearing (OR = 1.48, 95% CI = 1.08–2.02; *p* = 0.013) and memory problems (OR = 1.42, 95% CI = 1.00–2.02; *p* = 0.047), dementia (OR = 3.13, 95% CI = 1.79–5.46; *p* < 0.001) and hospitalizations of > 24 h in the last year (OR = 1.51, 95% CI = 1.04–2.20; *p* = 0.031) than healthy counterparts with strong muscle power. Overall, participants with poor lower-limb muscle power had a significantly higher risk of adverse events [in men: odds ratio (OR) = 1.51, 95% confidence interval (CI) = 1.19–1.91, *p* < 0.001; in women: OR = 1.52, 95% CI = 1.27–1.87, *p* = 0.001] than their stronger counterparts.

The seven percentile curves (P3 to P97) of lower-body muscle power (W·kg^−1^) for men and women are shown in Fig. 3.

## Discussion

Here, we have provided nationally representative estimates of gender-specific normative values for lower-body muscle power. Our study will aid in the clinical assessment of lower-body muscle power to help establish thresholds for identifying muscle weakness and monitoring relative muscle strength during aging in clinical and health-related settings.

To the best of our knowledge, this study is the first to examine the association between lower-body muscle power and disorders in older Colombian adults. Therefore, we determined the odds of adverse events for individuals with decreased lower-body muscle power according to the cut-off points found compared to their healthy counterparts. In this way, it was found that older male and female adults with low muscle strength according to the cut-off points had higher odds of adverse events in most of the disorders studied than their healthy counterparts. In numerical terms, of the 14 conditions presented, men had a statistically significant association with 7 and women with 9, respectively.

Our results showed a decrease in lower-limb relative power for both men and women, and it is noticeable that the decline is a trend across all ages and percentiles. The downward trend presents a similar pattern to that reported in a previous study using the same formula^22^. In contrast, our normative values are not comparable with other studies in which different methods were applied to assess lower-limb muscle strength^23,24^. Nevertheless, the results of this study contribute to updating the current body of literature because they show gender- and age-specific weakness cut-off points among the elderly Colombian population.

Normative values of relative power from a multicenter European study^22^ showed slightly higher values than our results: older Spanish, Belgian and Portuguese people had better performance on the STS test than our elderly Colombian people. These differences among countries are following studies that compared individuals from different geographic regions. For example Arokiasamy et al.^25^ reported that countries with better socio-economic status had better HGS than developing countries; Leong et al.^26^ showed that HGS values were highest among those from Europe/North America and lowest among those from South Asia, Southeast Asia and Africa. These discrepancies may be motivated by several factors, such as genetic, biological and environmental aspects (inequalities). Genetic characteristics and muscular performance have been studied, showing relative information about the differences among ethnicities^27^. However, the current miscegenation among different races, and therefore the genetic exchange between descendants, prevents us from asserting that the genetic factor is as decisive for muscle health as other factors; thus, future studies are needed^28^. Instead, we opted to partially explain the differences among countries by factors related to inequalities. In this sense, previous studies revealed a strong relationship between wealth and HGS^29,30^. Belonging to a high or medium socio-economic status allows access to a better health system and lifestyle. For example, individuals with a higher socio-economic status can perform more in-depth and continuous health checks, participate in physical exercise programs to improve health and have greater availability of foods rich in protein and vegetables. Unfortunately, access to these resources is limited in countries such as Colombia. Consequently, inequalities seem to be the main factor determining muscular weakness^31^.

In our study, we found associations between lower-limb muscle status and disorders. Generally, weaker individuals presented a statistically significant relationship with 7 of the 14 adverse events in men and 9 of the 14 adverse events in women. In line with our results, Steves et al.^32^ found that leg power predicts cognitive decline after 10 years in older female twins after controlling for physical activity, comorbidities, genetic characteristics and the environment shared by the twins. Canon and Crimmins^33^ showed that muscle quality of the knee extensors, assessed using an isokinetic device, was closely related to cognitive decline. Sverdrup et al.^34^ found an association with dementia for poor performances in the Timed Up and Go (TUG) test, the STS test and gait speed.

Additionally, several studies have related muscle weakness of the lower limbs with disability^35^ and falls^36^. Our muscle power cut-off points appear to have the ability to identify women hospitalized in the last year. In accordance with our results, Losa-Reyna et al.^37^ found that low relative muscle power was associated with a higher risk of hospitalization among 1928 older adults. Therefore, our findings reveal the importance of maintaining muscle strength to avoid losing functions related to neuromuscular and neurocognitive disorders and hospitalization.

The results of this study highlight the importance of evaluating lower body power using the STS test. However, the use of this test is not very widespread in clinical practice. We must bear in mind that the current guidelines for discriminating sarcopenia contemplate using HGS to assess strength. However, these guidelines do not apply the STS test. One possible reason why the test is not used could be the lack of standardization of the test and ignorance of how to transform the final result into quantifiable values relative to the subject's mass, such as those offered by the equation published by Alcazar et al.^8^ and used in this study. Instead, other methods have been used to quantify leg power, such as hand-held dynamometry^38^ and isokinetic dynamometry^39^. However, the STS muscle power test^8^ is an easy, inexpensive and feasible method that allows lower-limb muscle power to be assessed in a few minutes and at any location. Additionally, the STS muscle power test has been demonstrated to be valid for considering muscle power compared with assessments using linear position transducers^8^ and force platforms^40^.

The strengths of this study include its large sample size of older adults from a representative proportion of persons aged > 60 years. Additionally, the method used to assess muscle power can be used in future works and to compare the results with our normative values. However, the study has some limitations. Firstly, the muscle strength was estimated using a previously validated equation^8^ Secondly, the cross-sectional nature of our data may not allow a definitive conclusion that weak muscle strength precedes adverse events, and the results would not be comparable with those of longitudinal studies. Another potential limitation of this study could be the standardized use of a chair at the height of 45 cm. While it is true that a fixed chair height provides a standardized and reproducible field assessment in subsequent studies and waves, and the objective of this study was to establish normative data from a field test, the chair height could affect the results. In this sense, Nakamura et al. ^41^ compared the peak oxygen consumption, which increased as the seat height decreased. Kuo ^42^ found a direct relationship between chair height and performance in the 30-s STS test in community-dwelling older adults. In this study, the number of repetitions was improved on when the chair's height was increased and vice versa. Additionally, Yamada and Demura ^43^ measured the vertical floor reaction force and an electromyogram on the rectus femoris and tibialis anterior muscles during STS movement. They showed that the load and motor pattern selected by the individual differ because the seat height changes relative due to differences in thigh length. Thus, the chair height may affect the results, with more pronounced effects in individuals whose thigh length is short or exceeds the standardized height of the chair. Finally, our study presented a prevalence of 44% with poor gait speed and 65% with dynapenia, which are much higher values than those in the Health ABC study^43^ for example, so the results should be taken with caution. Unfortunately, data to test the validity of the detection algorithm (or of other algorithms) are not yet available.

## Conclusions

The present study provides reference values of lower-limb relative muscle power in a sample population consisting of 3689 Colombian older adults. We present gender-specific cut-off points for weak relative muscle power, showing that muscle power weakness was associated with a higher risk of neurocognitive and neuromuscular impairments, such as sarcopenia, dynapenia, low gait speed performance, cognitive impairment, mental, visual, hearing and memory problems, as well as dementia and hospitalization, compared with those attained by healthy counterparts.

## Supplementary Information

Supplementary Figure S1.

Supplementary Table S1.

Supplementary Table S2.

## Data Availability

The datasets generated during and/or analysed during the current study are available from the corresponding author on reasonable request.
