# Supplementary material for: Sit to stand muscle power reference values and their association with adverse events in Colombian older adults
Source: Sci Rep. 2022 Jul 12;12:11820. doi: 10.1038/s41598-022-15757-8 (PMC9276682; doi:10.1038/s41598-022-15757-8)
Supplement: Supplementary file 1 — Supplementary Figure S1. [file 41598_2022_15757_MOESM1_ESM.pdf]

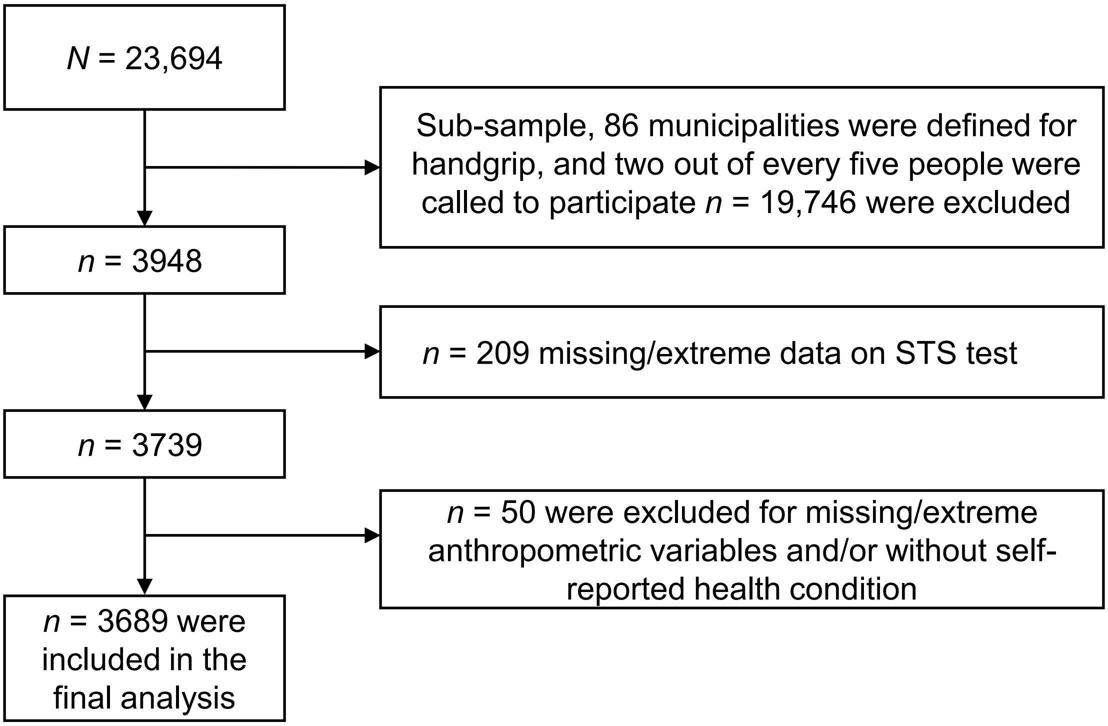


Figure S1. The flow chart shows the study sample selection from the Colombian Health and Wellbeing and Aging Survey (SABE) 2015. All analyses presented in this paper were based on 3689 surveyed participants, each with complete STS test and anthropometric data.
