# Supplementary material for: Sit to stand muscle power reference values and their association with adverse events in Colombian older adults
Source: Sci Rep. 2022 Jul 12;12:11820. doi: 10.1038/s41598-022-15757-8 (PMC9276682; doi:10.1038/s41598-022-15757-8)
Supplement: Supplementary file 2 — Supplementary Table S1. [file 41598_2022_15757_MOESM2_ESM.docx]

| **Supplemental Table S1**. Association between poor lower-limb muscle power (W·kg^-1^) with disorders and adverse events. Unadjusted analysis. | | | | | | | | | |  |  |
| --- | --- | --- | --- | --- | --- | --- | --- | --- | --- | --- | --- |
|  |  |  |  |  |  |  |  |  |  |  | |
|  |  |  |  |  |  |  |  |  |  |  | |
| **Disorders** | **Statistics for each variable** | | | | | | | | |  | |
|  | Odds ratio | Lower limit | Upper limit | p-Value |  | Odds ratio | Lower limit | Upper limit | p-Value |  |  |
| **MEN** | | | | |  | **WOMEN** | | | |  | |
| **Neuromuscular disorders** |  |  |  |  |  |  |  |  |  |  |  |
| Sarcopenia | 0.88 | 0.56 | 1.39 | 0.597 |  | 1.25 | 0.92 | 1.70 | 0.149 |  |  |
| Dynapenia | 1.88 | 1.42 | 2.49 | <0.001 |  | 2.70 | 2.10 | 3.46 | <0.001 |  |  |
| Poor gait speed | 3.24 | 2.29 | 4.57 | <0.001 |  | 2.72 | 1.88 | 3.91 | <0.001 |  |  |
| **Neurocognitive disorders** |  |  |  |  |  |  |  |  |  |  |  |
| Cognitive impairment | 1.70 | 1.14 | 2.54 | 0.009 |  | 1.90 | 1.40 | 2.58 | <0.001 |  |  |
| Mental problems | 2.05 | 1.26 | 3.35 | 0.004 |  | 1.45 | 1.02 | 2.07 | 0.038 |  |  |
| Visual problems | 2.01 | 1.43 | 2.83 | <0.001 |  | 1.04 | 0.79 | 1.38 | 0.748 |  |  |
| Hearing problems | 1.32 | 0.98 | 1.77 | 0.065 |  | 1.49 | 1.14 | 1.96 | 0.003 |  |  |
| Memory problems | 1.52 | 1.06 | 2.19 | 0.022 |  | 1.28 | 0.91 | 1.80 | 0.146 |  |  |
| Dementia | 2.16 | 1.50 | 3.13 | <0.001 |  | 1.51 | 0.85 | 2.67 | 0.154 |  |  |
| **Others disorders** |  |  |  |  |  |  |  |  |  |  |  |
| Hypertension | 0.92 | 0.70 | 1.23 | 0.598 |  | 1.05 | 0.83 | 1.34 | 0.652 |  |  |
| Diabetes | 0.85 | 0.56 | 1.28 | 0.442 |  | 1.37 | 1.02 | 1.84 | 0.033 |  |  |
| Cardiovascular | 0.98 | 0.64 | 1.50 | 0.952 |  | 1.08 | 0.77 | 1.52 | 0.662 |  |  |
| **Falls** | 1.23 | 0.88 | 1.68 | 0.195 |  | 1.06 | 0.83 | 1.36 | 0.611 |  |  |
| **Hosptalized >24h last year** | 1.19 | 0.87 | 1.64 | 0.258 |  | 1.03 | 0.81 | 1.32 | 0.776 |  |  |
