# Supplementary material for: Sit to stand muscle power reference values and their association with adverse events in Colombian older adults
Source: Sci Rep. 2022 Jul 12;12:11820. doi: 10.1038/s41598-022-15757-8 (PMC9276682; doi:10.1038/s41598-022-15757-8)
Supplement: Supplementary file 3 — Supplementary Table S2. [file 41598_2022_15757_MOESM3_ESM.docx]

| **Supplemental Table S2**. STS relative power cut point values using <1 SD by sex and age group. | | |
| --- | --- | --- |
| **Sex / age groups (years)** | | **Cut point (W·kg^-1^)** |
| **Men** |  |  |
| 60–64 |  | 1.78 |
| 65–69 |  | 1.67 |
| 70–74 |  | 1.51 |
| 75–79 |  | 1.42 |
| 80–84 |  | 1.29 |
| +85 |  | 1.01 |
| **Women** |  |  |
| 60–64 |  | 1.32 |
| 65–69 |  | 1.24 |
| 70–74 |  | 1.08 |
| 75–79 |  | 1.01 |
| 80–84 |  | 1.00 |
| +85 |  | 0.79 |
